# Neural Changes in Adolescents with Single Ventricle Congenital Heart Disease

**DOI:** 10.1007/s00246-025-04141-8

**Published:** 2026-01-06

**Authors:** Kamal R. Singh, Nancy A. Pike, Diana E. Kosoyan, Dineth R. Karunamuni, Nancy J. Halnon, Alan B. Lewis, Rajesh Kumar

**Affiliations:** 1 Departments of Anesthesiology and Perioperative Medicine, University of California Los Angeles, 56–141 CHS, 10833 Le Conte Ave, Los Angeles, CA 90095 USA; 2 Sue & Bill Gross School of Nursing, University of California Irvine, Irvine, CA 92697 USA; 3 Division of Pediatric Cardiology, University of California Los Angeles, Los Angeles, CA 90095 USA; 4 Division of Pediatric Cardiology, Children’s Hospital Los Angeles, Los Angeles, CA 90027 USA; 5 Radiological Sciences, University of California Los Angeles, Los Angeles, CA 90095-1763 USA; 6 Brain Research Institute, University of California Los Angeles, Los Angeles, CA 90095 USA

**Keywords:** Functional magnetic resonance imaging, Regional homogeneity, Resting-state, Cognition

## Abstract

Single ventricle heart disease (SVHD) adolescents show brain tissue injury in sites that mediate autonomic, mood, and cognition functions deficient in the condition, which may result from impaired neural interactions at resting state. However, it is unclear whether SVHD subjects have aberrant neural activities in those areas that can be examined with regional homogeneity (ReHo) measures, assessing local neural synchronization. We aimed to examine regional brain neural activity changes in SVHD compared to healthy controls using functional magnetic resonance imaging (fMRI). Resting fMRI data were collected from 27 SVHD and 31 controls using a 3.0-Tesla MRI scanner. Using the standard pre-processing steps, ReHo maps were calculated and transformed to z-scored maps, normalized to a common space, smoothed, and compared between groups (ANCOVA; qFDR corrected *p* ≤ 0.05; covariates: age and sex). Reduced ReHo appeared in brain sites, including the caudate, parietal cortex, frontal cortex, and amygdala, and increased ReHo emerged in the parietal cortex, insula, cerebellar vermis, hippocampus, para-hippocampal gyrus, cerebellar peduncles, and cerebellar cortex in SVHD over controls. SVHD adolescents show impaired neural synchronization at resting in areas involved in neurobehavior and cognition. The findings indicate the widespread impact on brain functional organization and may explain functional deficits observed in SVHD.

## Introduction

Single ventricle heart disease (SVHD) is a subgroup of complex congenital heart disease with over 50% showing evidence of neurocognitive deficits in school-age children and adolescents [1, 2]. Multiple autonomic deficits in SVHD include blood pressure issues, heart rate irregularities, and mood and cognition dysfunctions, including anxiety, depression, weaker pragmatic language, attention impairments, impulsiveness, and reduced executive functions [3, 4]. Brain tissue changes emerge in sites that mediate autonomic, mood, and cognition functions based on multiple magnetic resonance imaging (MRI) structural imaging, including gray matter evaluation, T2-relaxometry, and diffusion tensor imaging [5–8]. Structural changes appear in the caudate nuclei, hippocampus, insula, cingulate, prefrontal cortex, cerebellum, amygdala, thalamus and hypothalamus, mammillary bodies, and basal ganglia, anatomical sites that mediate in autonomic, mood, and cognition functions [9, 10]. However, it is unclear whether similar sites have altered neural activities at resting-state that can be examined with resting-state functional MRI.

Resting-state functional MRI can be used to evaluate spontaneous neural activities without any explicit task. This approach is useful to explore the normal brain’s functional organization and to examine alterations in neurological or psychiatric conditions. Several methods have been proposed to characterize different aspects of the resting-state brain activities, with the synchronization of distant brain regions usually measured with functional connectivity (FC) [11], while the local features of brain activity can be assessed by regional homogeneity (ReHo) [12]. Resting-state FC quantifies the synchronization of the blood oxygen level dependent (BOLD) time courses of distant brain regions by calculating correlation coefficients, and ReHo is a voxel-wise measure of the synchronization of the time courses of a certain region and its adjacent neighboring areas [12]. FC is more relevant to the network or global properties of the brain, and ReHo is dominated by the local properties of different brain regions. The ReHo methods are proposed based on the evidence from electrophysiology studies in which local neural activation would result in higher local field potentials that is linked with increased BOLD signal synchrony in a spatially-limited brain region [13]. Thus, higher ReHo reflects higher level of local neural activities and lower ReHo shows reduced local neural activities [12, 14]. This measure is more sensitive to local brain functional changes than other FC methods [12]. However, both increased and reduced ReHo values are considered to be abnormal neural activity that contributes to site-specific impaired brain function [15–20]. Therefore, ReHo methods may be helpful when examining resting-state functional changes in the SVHD population.

The purpose of this study was to examine regional brain neural activity changes in adolescents with SVHD compared to healthy controls using resting-state fMRI-based ReHo procedures. We hypothesized that brain sites that mediate autonomic, mood, and cognition functions will show altered ReHo in SVHD over healthy controls.

## Materials and Methods

### Subjects

Twenty-seven SVHD adolescents [age, 15.6 ± 1.4 years; 14 male; body mass index (BMI), 22.7 ± 5.7 kg/m^2^] and 31 healthy controls (age, 15.8 ± 1.4 years; 15 male; BMI, 22.9 ± 4.4 kg/m^2^) were studied between Feb 2016 - Sep 2019. SVHD participants were recruited through posters or practitioner recommendations from the University of California Los Angeles (UCLA) and Children’s Hospital Los Angeles (CHLA) pediatric cardiology clinics, and healthy participants were recruited from word of mouth or snowball sampling in the Los Angeles area. SVHD participants and controls were excluded if they were claustrophobic, had non-removable metal (e.g., braces, pacemakers), severe developmental delays that impact ability for self-reporting (e.g., cerebral palsy or serious hypoxic injury), preterm delivery (gestation less than 37 weeks), and use of extracorporeal membrane oxygenation (ECMO). Healthy controls were self-reported to have no chronic medical or mental health conditions, or prior incidents of head trauma. Informed consent was obtained from the parental and assent from the child if below the age of 18 years, and all subjects over 18 years of age signed the consent before participation in the study. The Institutional Review Boards at the University of California Los Angeles and Children’s Hospital Los Angeles approved the study protocol.

### Assessment of Mood Function

Two self-reported questionnaires, the Beck Anxiety Inventory (BAI) [21] and the Patient Health Questionnaire-9 (PHQ-9) [22], were used to examine anxiety and depression symptoms in all participants. The BAI has 21 multiple-choice items with each question scored between 0 and 3, for total scores ranging between 0 and 63. Anxiety symptoms were deemed to be present in the participant with a BAI score greater than 9 [21]. The PHQ-9 is a nine-item assessment of depressive symptoms, with each question scored between 0 and 3, with a total score ranging between 0 and 27. Minimal, moderate, moderately-severe, and severe depressive symptoms were indicated for scores ranging from 5 to 9, 10–14, 15–19, and greater than 20 [22].

### Cognition Evaluation

The Montreal Cognitive Assessment (MoCA) test was used to examine cognition in both SVHD and control participants. This test examines several cognitive domains, including executive function, language, memory, visuospatial skills, conceptual thinking, attention and concentration, computations, and orientation [23]. The total score ranges from 0 to 30 with scores less than 26 deemed abnormal. The MoCA has been validated in adolescence age 14 and up with and without congenital heart disease [24].

### Magnetic Resonance Imaging

Brain MRI scans were collected when individuals were lying supine using a 3.0-Tesla MRI scanner (Magnetom Prisma-Fit, Erlangen, Germany). Foam pads were used on both sides of the head to restrict head movements. Resting-state functional MRI data were collected with interleaved ascending order using an echo planar imaging (EPI) sequence (repetition time [TR] = 2000 ms; echo-time [TE] = 30 ms; flip angle [FA] = 90˚; field-of-view [FOV] = 230 × 230 mm^2^; matrix size = 64 × 64; slice thickness = 4.2 mm; voxel size = 3.59 × 3.59 × 4.2 mm^3^; volumes = 100). Proton-density (PD) and T2-weighted images were obtained using a dual-echo turbo spin-echo sequence in the axial plane (TR = 10,000 ms, TE1 = 17 ms, TE2 = 134 ms, FA = 130°, matrix size = 256 × 256, FOV = 230 × 230 mm², and slice thickness = 3.5 mm). High-resolution T1-weighted images were also collected using a magnetization prepared rapid acquisition gradient-echo (MPRAGE) pulse sequence (TR = 2200 ms; TE = 2.41 ms; inversion time = 900 ms; FA = 9˚; matrix size = 320 × 320; voxel size = 0.72 × 0.72 × 0.9 mm^3^; FOV = 230 × 230mm^2^; slice thickness = 0.9 mm; number of slices = 192).

## Data Processing

### Visual Examination

Both high-resolution T1-weighted and proton-density and T2-weighted images were visually-examined for any anatomical defects before data processing. Resting-state functional MRI data were also examined for any scanning related artifacts. No significant brain injuries, head motions, or other imaging artifacts emerged in any of the participants included here.

### Resting-State Functional MRI Data Preprocessing

We used the DPARSFA (http://rfmri.org/DPARSF) and SPM12 (https://www.fil.ion.ucl.ac.uk/spm) software to preprocess resting-state fMRI data. Since temporal misalignment across slices was negligible and unlikely to have a substantial impact on ReHo analyses due to short TR and interleaved data acquisition, slice-timing correction was not performed [25, 26]. The first 10 brain volumes were removed to prevent signal saturation problems, and the remaining 90 brain volumes were realigned to remove any possible head-motion. The denoising step included regression of nuisance covariates, specifically Friston-24 head motion parameters, white matter, and cerebrospinal fluid (CSF) signals, followed by temporal band-pass filtering (0.01–0.08 Hz). In the nuisance regression step, DPARSFA utilizes a discrete cosine transform basis set to model low-frequency fluctuations; 128 s cut-off (approximately 60 basis functions) was used as default. We used nonlinear transformation techniques to spatially-normalize images to Montreal Neurological Institute (MNI) space. High-resolution T1-weighted from all SVHD and control participants were also normalized to MNI space and averaged. The mean anatomical image was used as a background for brain sites identification.

### ReHo Analysis

After data preprocessing, the DPARSFA toolbox was used to compute whole-brain ReHo maps. The Kendall’s coefficient of concordance between the voxel’s fMRI time course and that of its 26 closest neighbors was used to calculate the ReHo value for each voxel across the brain. Whole-brain ReHo maps were transformed into z-scored maps using Fisher’s z transformation to enhance normality and make statistical analyses more reliable. Whole-brain z-transformed maps were smoothed using a 4 mm Gaussian kernel with DPARSFA. Since smoothing before ReHo calculation may influence the resting-state fMRI signal and affect regional ReHo values, smoothing was performed after ReHo calculation and converting to whole-brain z-transformed maps as recommended by others [18, 27–30].

### Statistical Analysis

The IBM statistical package for the social sciences (IBM SPSS, v 30, Chicago, IL) was used for data analysis. Demographic and clinical values were assessed with independent samples t-tests and categorical characteristics with the Chi-square. A *p* < 0.05 value was considered statistically significant. For examining ReHo differences between groups, the normalized and smoothed whole-brain ReHo maps were compared between SVHD and controls using analysis of covariance (ANCOVA), with age and sex as covariates (SPM12). To correct for multiple comparisons, a voxel level adjusted false discovery rate (qFDR) correction was applied with a threshold of q ≤ 0.05. The statistical parametric maps showing brain sites with significant ReHo differences between groups were superimposed onto the mean anatomical image for brain regions identification. We also performed voxel-wise partial correlations between ReHo values and clinical scores (MoCA, BAI, and PHQ-9) in SVHD subjects, accounting for age and sex.

## Results

### Demographic and Clinical Characteristics

Demographic, physical, and clinical variables of SVHD and controls are summarized in Table 1. No significant differences in age (*р*=0.624), sex (*р*=0.792), body mass index (*р*=0.891), ethnicity (*р*=0.965), or handedness (*р*=0.590) appeared between groups. SVHD adolescents showed significantly higher BAI scores (*p* = 0.011), but not PHQ-9 scores (*p* = 0.148) compared to controls. Global MoCA scores and majority of sub-scores were significantly lower in SVHD compared to controls (*p* < 0.05).

**Table 1:** Demographic, biophysical, and clinical data of SVHD and control subjects

Variables	SVHD (mean ± SD) [*n* = 27]	Controls (mean ± SD) [*n* = 31]	*p* values
Age range (years)	14–18	14–18	−
Mean age (years)	15.59 ± 1.37	15.77 ± 1.43	0.624
Sex (Male: Female)	14:13	15:16	0.792
Ethnicity (White: Hispanic: Other)	8:15:4	10:17:4	0.965
BMI (kg/m^2^)	22.7 ± 5.74	22.88 ± 4.4	0.891
Handedness (Right: Left: Ambidextrous: Unknown)	21:5:1:0	25:3:2:1	0.590
Ventricle type (Right: Left: Unknown)	16:6:5	−	−
Fenestration (yes: no: N/A)	7:14:6	−	−
PHQ-9	5.93 ± 4.09	4.45 ± 3.57	0.148
BAI	17.96 ± 12.58	10.26 ± 9.65	**0.011**
Total MoCA scores	22.33 ± 4.57	27.94 ± 2.18	**< 0.001**
MoCA: Visuospatial	3.52 ± 1.34	4.52 ± 0.996	**0.002**
MoCA: Naming	2.85 ± 0.362	3.00 ± 0.00	**0.026**
MoCA: Attention	4.07 ± 1.47	5.32 ± 1.14	**< 0.001**
MoCA: Language	1.52 ± 1.12	2.61 ± 0.56	**< 0.001**
MoCA: Abstraction	1.07 ± 0.68	1.84 ± 0.37	**< 0.001**
MoCA: Delayed Recall	2.41 ± 1.69	3.87 ± 1.38	**< 0.001**
MoCA: Orientation	5.81 ± 0.68	5.90 ± 0.396	0.542

*SD* Standard deviation, *N/A* Not applicable, *PHQ-9* Patient Health Questionnaire-9, *BAI* Beck Anxiety Inventory, *BMI* Body mass index, *MoCA* Montreal Cognitive Assessment. Significant p-values are highlighted in bold

### Resting-State ReHo Changes

**Fig. 1:**
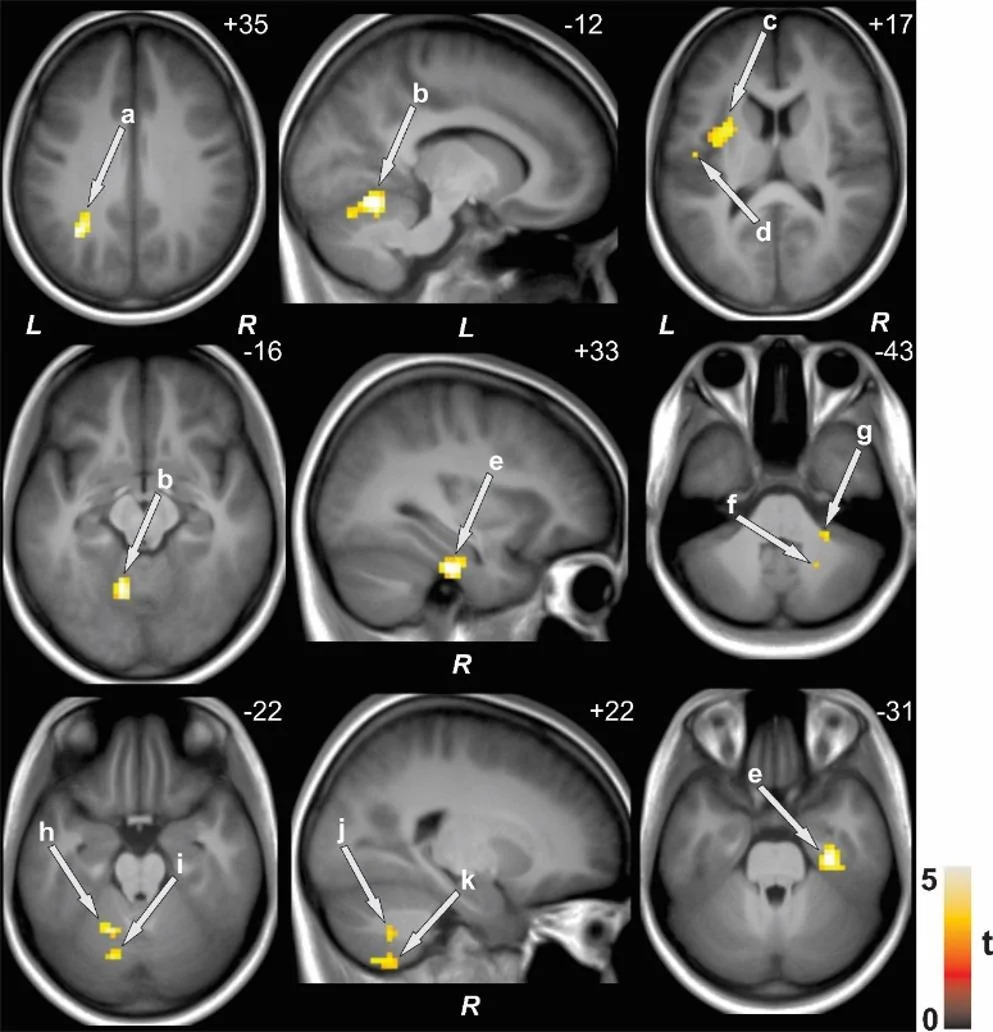
Brain sites with increased ReHo in SVHD compared to healthy controls. Brain regions with increased ReHo were observed in the left parietal cortex (**a**), left anterior and mid insula (**d**, **c**), left cerebellar vermis (**b**, **h**, **i**), right hippocampus (**e**), extending to para-hippocampal gyrus (**e**), right inferior cerebellar peduncles (**f**, **g**, **j**), and right cerebellar cortex (**k**) in SVHD compared to healthy controls. All images are in neurological convention (L = Left; R = Right). Color bar indicates t-statistic values, with black/red color shows less significance and yellow color indicates higher significance in ReHo among SVHD and healthy controls

**Fig. 2:**
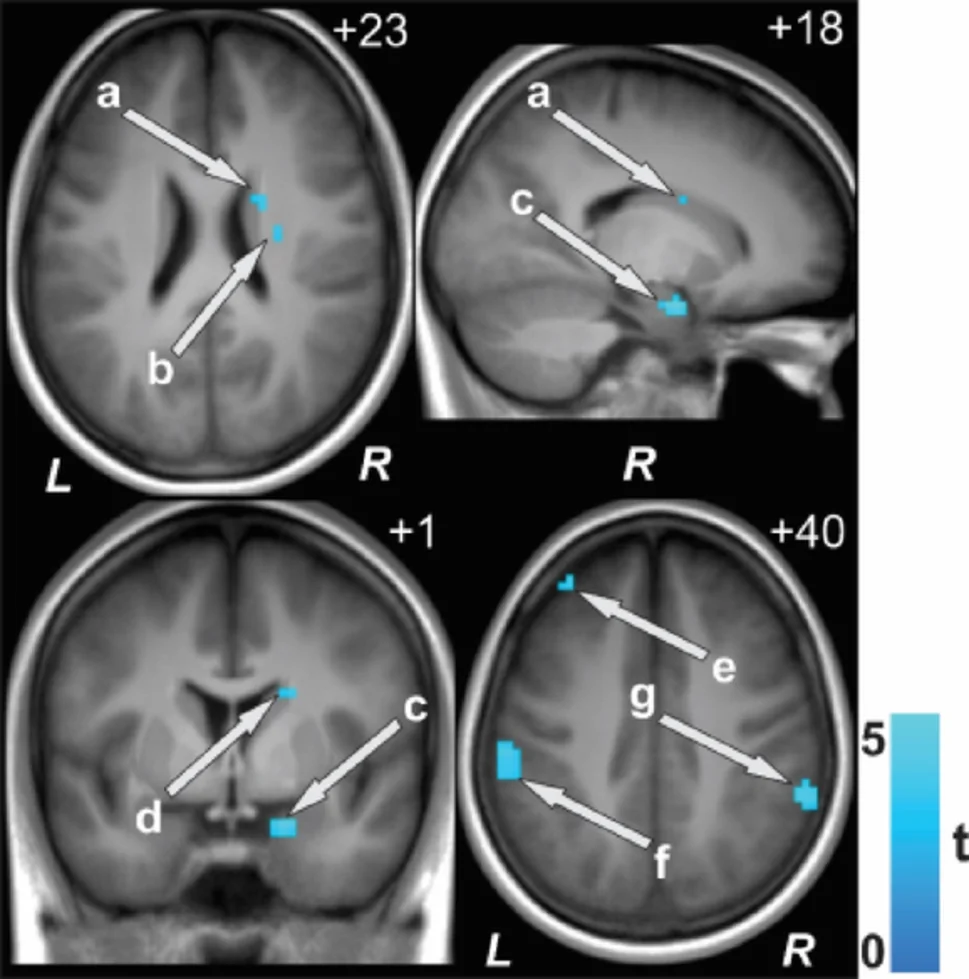
Brain sites with decreased ReHo in SVHD compared to healthy controls. Brain regions with decreased ReHo were observed in the right caudate (**a**, **b**), bilateral parietal cortices (**f**, **g**), left frontal cortex (**e**), right frontal white matter (**d**), and right amygdala (**c**) in SVHD compared to healthy controls. All images are in neurological convention (L = Left; R = Right). Color bar indicates t-statistic values, with medium blue color shows less significance and bright cyan color indicates higher significance in ReHo among SVHD and healthy controls

After accounting for age and sex, several brain regions showed increased ReHo in SVHD participants compared to healthy controls. In addition, some brain sites showed decreased ReHo in SVHD participants over healthy controls. Brain areas with increased ReHo values in SVHD emerged in the left parietal cortex (a), left anterior and mid insula (d, c), left cerebellar vermis (b, h, i), right hippocampus (e), extending to para-hippocampal gyrus (e), right inferior cerebellar peduncles (f, g, j), and right cerebellar cortex (k) compared to healthy controls (Fig. 1; Table 2). Brain sites with decreased ReHo emerged in the right caudate (a, b), bilateral parietal cortices (f, g), left frontal cortex (e), right frontal white matter (d), and right amygdala (c) in SVHD participants over healthy controls (Fig. 2; Table 3).

**Table 2:** Coordinates of brain sites with increased ReHo in SVHD compared to healthy controls

Brain area	Coordinate	q_FDR−corr_
Left parietal cortex (a)	(a: [−33, −58, 35])	0.034
Left anterior and mid insula (d, c)	(d: [−48, −22, 20], [−45, −10, 20); (c: [−33, −1, 17])	0.019
Left cerebellar vermis (b, h, i)	(b: [−12, −61, −16], [−15, −73, −22]); (h: [−17, −57, −23]); (i: [−13, −71, −23])	0.013
Right hippocampus (e), extending to para-Hippocampal gyrus (e),	(e: [33, −22, −31], [48, −25, −31], [54, −34, −31])	0.004
Right inferior cerebellar peduncles (f, g, j)	(f: [21, −58, −43]); (g: [27, −46, −46]); (j: [22, −58, −43])	0.050
Right cerebellar cortex (k)	(k: [24, −55, −61], [18, −64, −58])	0.021

**Table 3:** Coordinates of brain sites with decreased ReHo in SVHD compared to healthy controls

Brain area	Coordinate	q_FDR−corr_
Right caudate (a, b)	(a: [20, 2, 23]); (b: [27, −10, 26])	0.050
Bilateral parietal cortices (f, g)	(f: [−30, 47, 35]); (g: [57, −43, −44], [60, −37, 35])	0.050 0.050
Left frontal cortex (e)	(e: [−34, 42, 40])	0.050
Right frontal white matter (d)	(d: [19, 1, 23])	0.027
Right amygdala (c)	(c: [18, −1, −22])	0.050

### ReHo Values and Clinical Scores

**Fig. 3:**
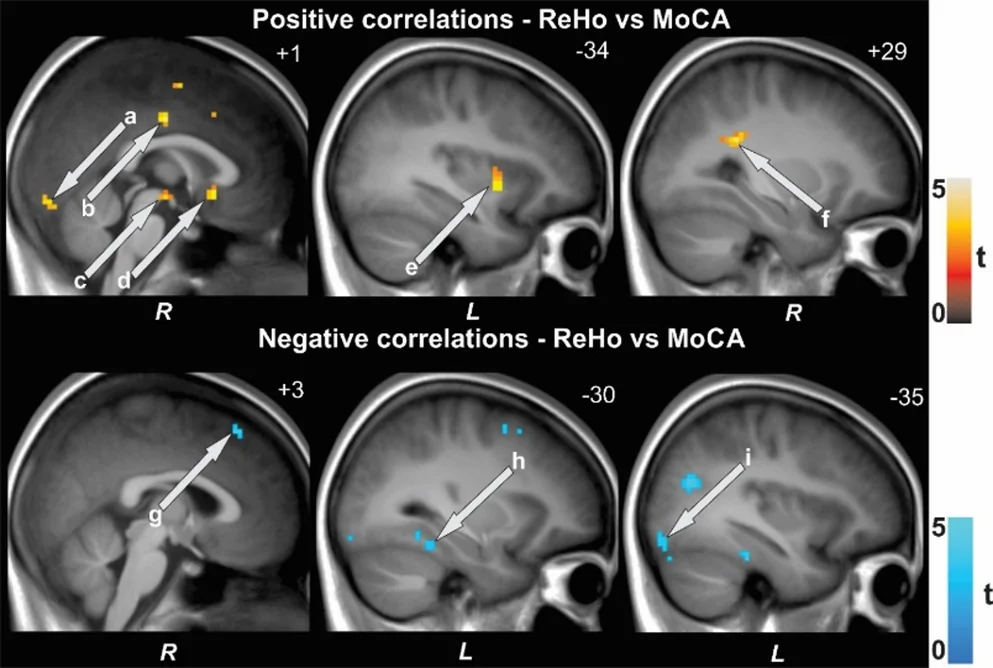
Brain sites showing associations between ReHo and MoCA in SVHD. Using a voxel-wise threshold of *p* < 0.005, cerebellar vermis (**a**), anterior and mid cingulate cortices (**b**), mid brain (**c**), right anterior cingulate (**d**), left anterior insula (**e**), right temporal white matter region (**f**), showed significant positive correlation, and medial prefrontal cortex (**g**), left para-hippocampal gyrus (**h**), left occipital cortex (**i**) showed significant negative correlations between ReHo and MoCA

**Fig. 4:**
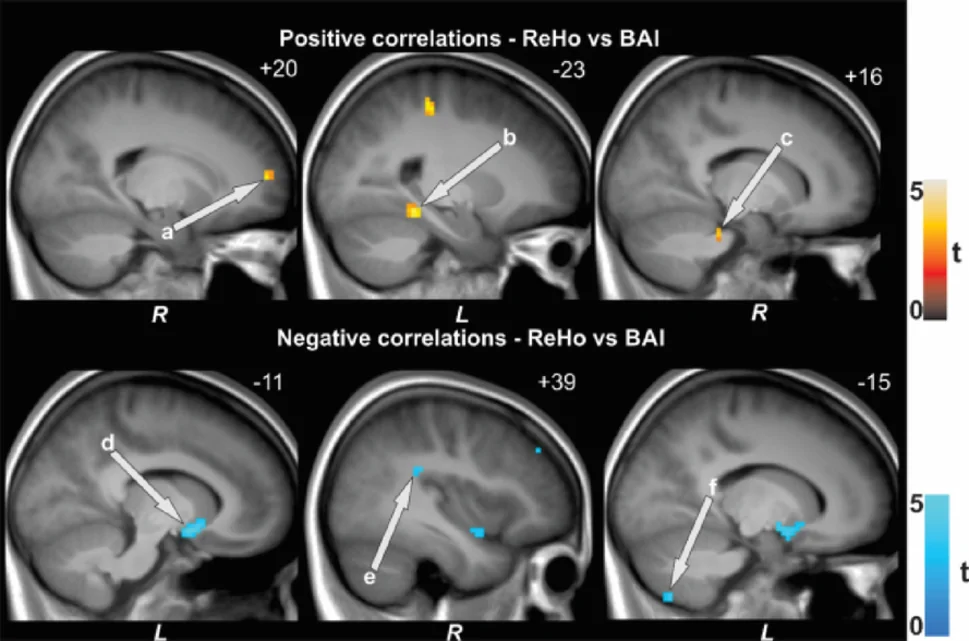
Brain sites showing associations between ReHo and BAI in SVHD. Using a voxel-wise threshold of *p* < 0.005, frontal cortex and white matter regions (**a**), left para-hippocampal gyrus (**b**), right cerebellar peduncle (**c**), showed significant positive correlations, and left caudate (**d**), right posterior insula (**e**), left cerebellar cortex (**f**) showed negative correlations between ReHo and BAI

**Fig. 5:**
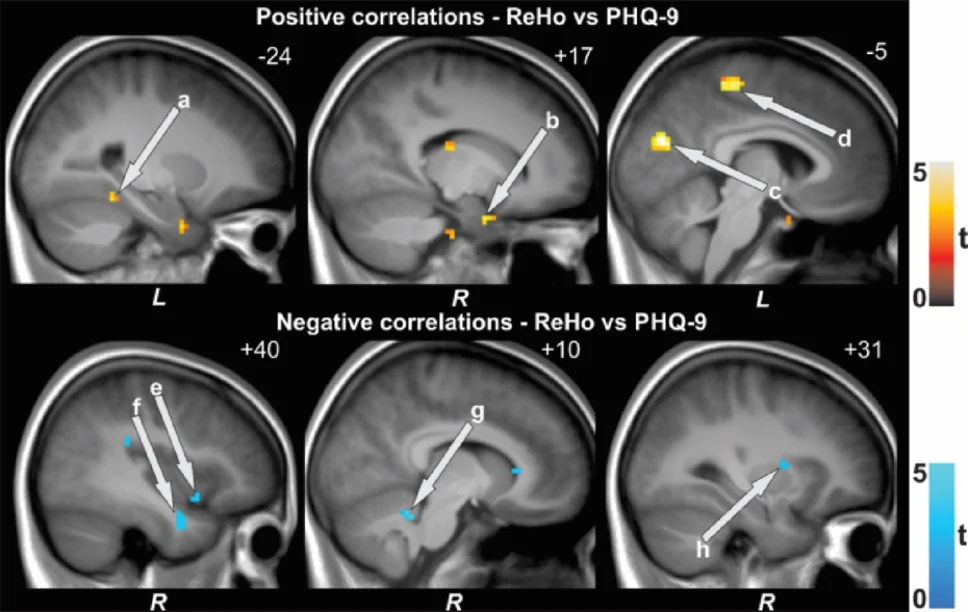
Brain sites showing associations between ReHo and PHQ-9 in SVHD. Using a voxel-wise threshold of *p* < 0.005, left para-hippocampal gyrus (**a**), right amygdala (**b**), middle occipital cortex (**c**), left parietal cortex (**d**), showed positive significant correlations, and right anterior insula (**e**), right temporal white matter (**f**), cerebellar vermis (**g**), right globus pallidus (**h**), showed negative correlations between ReHo and PHQ-9

After accounting for age and sex, several brain sites showed positive or negative correlations between ReHo and MoCA in SVHD (Fig. 3; *p* < 0.005). Positive correlations appeared in the right anterior and mid cingulate cortices (d, b), cerebellar vermis (a) and cortical areas, midbrain (c), left anterior insula (e), ventral temporal cortex, and right temporal white matter regions (f), and significant negative correlations emerged in the left prefrontal and medial prefrontal cortices (g), left occipital cortex (i), left para-hippocampal gyrus (h), left partial cortex, and right frontal region between ReHo and MoCA. However, none of these sites survived for multiple comparisons (qFDR correction; *p* < 0.05). Brain sites, including the parietal cortex and surrounding white matter, right prefrontal and frontal cortices and white matter regions (a), right temporal cortex, right anterior insula extending to white matter, left para-hippocampal gyrus (b), and right cerebellar cortex and peduncles (c) showed significant positive correlations, and left parietal cortex, bilateral prefrontal cortices, dorsal temporal cortex, left caudate (d) and putamen, right posterior insula (e), and left cerebellar cortex (f) showed negative correlations between ReHo and BAI values (Fig. 4). No clusters in these areas survived for multiple comparisons (qFDR correction; *p* < 0.05). Also, brain areas including the bilateral parietal cortices (d), right medial prefrontal cortex, middle occipital cortex (c), left anterior thalamus, right frontal white matter, left para-hippocampal gyrus (a), and right amygdala (b) showed positive correlations, and left prefrontal cortex, bilateral parietal cortices, bilateral anterior insular cortices (e), left occipital cortex, left caudate and internal capsule, bilateral globus pallidus (h), right temporal white matter (f), and cerebellar vermis (g) showed negative correlations between ReHo and PHQ-9 scores (Fig. 5). Correction for multiple comparisons showed no significant correlations between ReHO values of these areas and PHQ-9 scores (qFDR correction; *p* < 0.05).

## Discussion

Adolescents with SVHD showed both increased and decreased ReHo changes at resting-state in multiple cortical and subcortical areas which are crucial sites that mediate emotion, autonomic, and cognition regulations. These brain functions have been previously reported to be compromised in the condition. Similar brain regions showed structural brain injury in SVHD [5–8] indicating damage in those areas responsible for these functions. The findings indicate that aberrant neural synchronization at resting-state may contribute to functional deficits in the condition with presence of both hypo- and hyper-synchronization in particular brain areas.

### Increased and Decreased ReHo

Multiple cortical and subcortical brain sites emerged with increased and decreased ReHo values in SVHD adolescents. Both increased and decreased ReHo values are considered as functional deficits [26], and several disease conditions, including Parkinson’s disease [25], schizophrenia [31], major depressive disorder [32], and attention deficit hyperactivity disorder [33] show altered ReHo values. Decreased ReHo indicates that neural activity in certain brain areas is less synchronized, which may result from neural injury in those sites as observed in SVHD adolescents. Multiple neuroimaging studies have shown that children and adolescents with SVHD exhibit structural changes in brains regions, including altered cortical development, subcortical atrophy, reduced gray matter volume, and white matter damage [10]. However, an increased ReHo may result from excessive neuronal activity in these brain areas as a compensatory effect, and such effects have been found in other conditions with hypoxia/ischemic injury.

### SVHD and Structural Changes

Several studies indicate common brain structural changes in SVHD adolescents and suggest that chronic hypoxemia and decreased cerebral blood flow over childhood may be the major causes [34]. Such neural injuries are localized in the hippocampus, insula, cingulate, caudate, putamen, cerebellar cortex, amygdala, and other major regions. Although injured regions are predominantly watershed, the hippocampus is highly susceptible to hypoxia-ischemia, regularly exhibits substantial volume decreases in SVHD [5, 6]. Impairments in executive function, general memory, and delayed verbal recall are associated with hippocampal changes. In addition, cerebellar cortices are sensitive to hypoxia/ischemia, especially Purkinje cell. The cerebellum is involved in both motor and cognitive function, and cerebellar volume loss is linked to poorer executive ability, processing speed, and inhibitory control [5]. These results imply that structural anatomical changes in the hippocampus and cerebellum play a major role in the cognitive impairments reported in previous SVHD studies.

Another SVHD characteristic may include cortical maldevelopment/dysmaturation, which goes beyond regional volume reduction [35] and may include changes in tissue diffusion characteristics. Decreased sulcal pattern homogeneity, especially in the temporal and parietal lobes, have been linked in structural studies to poorer executive functioning, speed of processing, and impaired intelligence. In addition, significant tissue microstructural impairments in gray and white matter, such as decreased fractional anisotropy and increased mean diffusivity in key regions have also been observed by DTI. Such findings imply compromised axonal integrity and myelination [36] that may result from maldevelopment or hypoxemic effects.

### ReHo Changes in SVHD

Brain areas with decreased ReHo in SVHD compared to health controls appeared in the caudate, parietal cortex, frontal cortex and nearby region, and amygdala, sites that are linked mediating attention, motor control, executive function, and emotion. Multiple higher cognition functions, including motivation, emotion, learning, memory, and executive functioning [37, 38] are depend on the caudate nuclei, and such decrease in ReHo may suggest a functional disorder in cortico-striatal circuits, leading to cognition deficits. Both parietal and frontal cortices, which showed lower ReHo values, are important regions of the fronto-parietal control system [8]. Higher-order cognition processes are regulated from these areas, and executive dysfunction and attentional deficiencies frequently observed in SVHD may result from compromised neuronal synchronization. In addition, the amygdala showed reduced ReHo changes that plays critical roles in processing and regulating emotions, and affective symptoms in SVHD may contributed from this site of the affective circuitry.

Interestingly, SVHD participants showed increased ReHo in a number of brain regions, including the insula, cingulate, parietal, hippocampus and para-hippocampus gyrus, and cerebellar areas. Parietal cortex is involved integrating sensory information and visuospatial attention as alternative area, when broad functional sensory connection are impaired [39]. The insula, an important site for autonomic and mood regulation, salience identification, and interoceptive perceptions, also displayed increased ReHo, especially at the anterior and mid regions. These findings are consistent with earlier results showing the role of insula in autonomic dysfunction in SVHD adolescents. A rise in local coherence was observed in the cerebellar vermis and cortical sites that are involved in executive functioning and emotion regulation [40]. Both the right hippocampus and the para-hippocampal gyrus, which are crucial for spatial processing, mood, and memory encoding, showed increased ReHo, and such compensatory effects may contribute to mood and cognition impairment in SVHD.

### Correlations Between ReHo Values and Clinical Scores

Multiple brain areas showed either positive or negative correlations between ReHo values and clinical scores, including the MoCA, BAI, and PHQ-9 values, which may have resulted from significantly increased or decreased ReHo values in those areas. Such correlations emerged with uncorrected statistical threshold, but did not survive for multiple comparison corrections, indicating weak correlations, probably resulting from small sample size. Brain sites with correlations between ReHo and MoCA values indicate involvement in cognition processing in SVHD adolescents. Positive correlations between ReHo and MoCA values in the temporal and cingulate regions suggest that such networks can facilitate semantic processing and cognitive control either with compensatory or maintained integration. Negative correlations between ReHo and MoCA scores in SVHD subjects that emerged in the insular, cerebellar, occipital, and frontal regions, on the other hand, can indicate deficiencies or changed coupling in networks related to higher-order executive tasks, salience appraisal, and sensorimotor coordination [41, 42]. Anxiety symptoms in SVHD may be associated with decreased or hyper local synchronization across a diffuse mix of sensory and affective networks, as found here (Fig. 4). Increased local neural synchronization among circuits associated with affective-somatosensory integration is indicated by positive correlations appeared in the cerebellar, occipital, putamen, and temporal sites, areas that are involved in anxiety symptoms in multiple cardiovascular and neurological disorders. In addition, negative correlations in insular sub-divisions may indicate a lack of functional coordination within the salience–interoceptive network, responsible for threat detection, interoceptive consciousness, and anxiety-induced physiological reactions [43, 44]. Depressive symptoms in SVHD may be associated with changes in local connections in sensorimotor and affective-related brain areas. Several brain areas showed positive and negative correlations between ReHo and PHQ-9 values in SVHD adolescents (Fig. 5). Negative correlations in the frontal and insular areas may suggest reduced local processing in networks related to regulating emotions, reward, and interoceptive perception, and positive correlations in sensorimotor cortices and cerebellar sites could indicate hyper regional synchronization linked to somatic/behavioral depression aspects [45–47].

### Clinical Implications

Treatment for aberrant neural synchronization is often a multifaceted approach focused on modulating neural activity to restore healthy asynchronous firing patterns or promote synchronized activity that is beneficial for cognition function. Other approaches are pharmacologic medications that impact neuromodulators, including dopamine and serotonin, and/or neurofeedback and behavioral techniques to self-regulate brain activity and enhance interpersonal neural synchrony through social activities. Future research is needed to assess interventions to improve cognitive function utilizing neural synchronization techniques in the SVHD population.

### Limitations

It is worthwhile to recognize that this study has multiple limitations, including small sample size, limited participants based on ventricle types, small length of fMRI series, and inclusion of SVHD adolescents with better health. Although this study had a small sample size, substantial group differences appeared in multiple brain sites, indicating a high effect size between SVHD and controls. SVHD adolescents show differential structural and hemodynamic changes [48] based on single left or right ventricle. However, due to our small sample size, we were unable to perform analysis based on ventricle type. Also, correlations between ReHo values and clinical scores did not survive for multiple comparisons in SVHD adolescents that may have resulted due to small samples. Additionally, these results could not be generalized to all SVHD adolescents, since the sample selection was bias toward participants that might have better health with not having a pacemaker, born preterm, or having other co-morbidities. In addition, since this study was conducted on adolescents, the resting-state fMRI acquisition time was thoughtfully kept small to minimize potential head-motion that may have reduced statistical power to identify additional brain area differences between SVHD and healthy controls.

## Conclusions

SVHD adolescents show significant regional impaired neural activity at resting-state in brain areas that mediate in multiple neurobehavioral and cognition functions, which are commonly found deficient in the condition. Impaired neural activity in SVHD emerged as increased and decreased neural synchronization at resting-state in cortical and sub-cortical regions involved in sensorimotor, cognition, emotional, and autonomic regulation, reflected as aberrant functional deficits. The functional deficits in SVHD adolescents may result from delayed brain development and/or hypoxia/ischemia-induced neural changes in the condition.

## Data Availability

The processed datasets generated during and/or analyzed in the current study are available from the corresponding author upon reasonable request.
